# Analysis of inequality in the distribution of general practitioners in China: evidence from 2012 to 2018

**DOI:** 10.1017/S1463423622000408

**Published:** 2022-09-19

**Authors:** Le Yang, Jingmin Cheng

**Affiliations:** School of Management, Shanxi Medical University, Taiyuan, Shanxi Province, China

**Keywords:** China, distribution, general practitioner, inequality

## Abstract

**Aim:**

This paper aims to analyze the inequalities in general practitioner (GP) distribution in China.

**Background:**

GPs-based primary health care (PHC) has been implemented from 2011 in China, aiming to improve the accessibility and quality of basic medical and healthcare services. GPs in China, as the gatekeeper of people’s health, mainly undertake integrated health services at the grass-roots level.

**Methods:**

The number of GPs and inequality in GPs distribution from 2012 to 2018 was analyzed by the Lorenz Curve/Gini coefficient and Theil L index. Data were extracted from *China Health Statistical Yearbook 2013–2019*.

**Findings:**

The demographic Gini coefficient of GPs changed from 2012 (0.234) to 2018 (0.167), showing high equality in China. In contrast, the Thiel L index from 2012 (0.372) to 2018 (0.345) showed less equality. The decomposition of Thiel L index implicated the inequalities within the divisions. The number of GPs in China shows a fast growth trend since the general practice system established, and the GPs distribution becomes more demographically equitable. However, the shortage of GPs and inequality in their distribution remains severe. More incentive and supportive policies need to be made to enhance the quantity, quality, and structure of GPs in China.

## Introduction

Primary health care (PHC), as a significant role in protecting and improving people’s health and decreasing healthcare costs, has been attached to high importance by many countries (Shi *et al.*, 2013; Phillips *et al.*, 2014; Yang and Wang, 2019). The inequalities in high-quality health service supply, especially in PHC, remained a serious challenge to China’s healthcare system, as well as the whole world (Gao *et al.*, 2002; Wang, Wang, and Maitland, 2012; Chen *et al.*, 2019). In China, the gap between supply and demand, urban and rural areas, the Eastern area and the Western area, and the health institutions in the grass-roots level and tertiary hospitals in health service has always been the great obstacles to promoting and protecting the health of the whole nation (Wang, Wang, and Maitland, 2012; Wu *et al.*, 2017; Yang, Wang, and Xue, 2019). The utilization of PHC in China is low (Zou *et al.*, 2015), and patients prefer to tertiary comprehensive hospitals for high-quality health service and medical technology (Jiang *et al.*, 2015), which causes the misuse of the medical resources, higher medical costs under the medical reimbursement policy, and the overload in the tertiary comprehensive hospitals leading to more medical conflicts and lower satisfaction. Moreover, the physicians in the grass-roots level were more likely to quit and find positions in big hospitals to obtain better career development and job satisfaction, further weakening the poor PHC system (Gan *et al.*, 2018).

To address the inequalities in health resources, the Chinese government has made great efforts in strengthening the PHC system and increasing the investments in the health institutions at the grass-roots level since the new health system reform launched in 2009 (Li *et al.*, 2020; Tao *et al.*, 2020). The *Opinions of the Communist Party of China (CPC) Central Committee and the State Council on Deepening the Health Care System Reform* issued in 2009 laid stress on the significant improvement of the accessibility of basic medical and healthcare services and effective reduction of the burden of residents’ medical expenses. At that time, the gatekeeper system in health care had been established, which was proved as an effective system to reduce medical cost, enhance medical efficiency, and provide convenient and accessible healthcare service to the public (Reibling and Wendt, 2012; Liu *et al.*, 2019). However, the underuse of PHC service had not been effectively improved as the government expected (Chen *et al.*, 2019). To reverse this situation, the Chinese government issued the General Practice System in 2011, which attempted to cultivate and recruit more practitioners and reserve patients at the grass-roots level health institutions under the tiered diagnosis and treatment model, implementing the PHC system based on general practitioner (GP) contract service (The Central People’s Government of the People’s Republic of China, 2011).

Compared with the specialist, GPs, as the gatekeepers of people’s health, provide comprehensive PHC at the grass-roots level, and patient with severe illness should first seek GP service and then be referred to a larger hospital. As the General Practice System guided, most Chinese GPs worked at the grass-roots level health institutions, such as community health centers, community health stations, township health centers, village clinics, and other PHC institutions (The Central People’s Government of the People’s Republic of China, 2011; Hung *et al.*, 2013). To alleviate the shortage of PHC workforce, especially in rural areas, the Chinese government made various GP training programs (Table 1), including the “5 + 3 program,” “3 + 2 program,” and “transition training program of rural specialists” (Cao *et al.*, 2014). GPs in China, trained to have a high degree of comprehensive medical knowledge and skills, mainly undertake integrated services such as preventive health care, diagnosis, and treatment of common and frequently occurring diseases and referral of patients with severe and intractable diseases, patient rehabilitation and chronic disease management, and health management at the grass-roots level. The “5 + 3 program” is the common training for GPs, consisting of five years of undergraduate clinical medicine education and three years of GPs standardized training or three years of postgraduate education. The “3 + 2 program” mainly trains the assistant GPs directed to the rural and remote areas with three years of medical education in medical college and two years of residency and public health training. The transition training program of rural specialists is not less than 12 months and can be completed within two years, and includes theoretical training (not less than one month), clinical skill training (not less than 10 months), primary care practice (not less than one month), and GP clinical thinking training (not less than 20 class hours) (Cui *et al.*, 2016; National Health Commission of the People’s Republic of China, 2019a). The rural specialists, who have passed the qualification examination for licensed (assistant) doctors, can get the certificate of specialists’ transition to GPs and register as a GP or an assistant GP in their local places after the training and passing the exams. And in 2015, China established the rural assistant GP system, adding the qualification examination for rural assistant GP to the current qualification examination for licensed assistant doctors. The rural specialists can register as an assistant GP in the township health centers or village clinics after passing the qualification examination for rural assistant GP. In this study, all GPs, either those who obtain a certificate of qualification after standardized training of GPs or licensed doctors who have registered in general practice, were included in the analysis.

**Table 1.** GP training system in China

Licensed GP		Licensed-assistant GP	
Medical students	Rural specialists (license doctor)	Medical students	Rural specialists
“5 + 3 program” five years of undergraduate clinical medicine education and three years of GPs standardized training or the three years of postgraduate education	Transition training program, not less than 12 months and can be completed within two years	“3 + 2 program” three years of medical education in medical college and two years of residency and public health training	Rural specialists (assistant license doctor): not less than 12 months and can be completed within two years
			Rural specialists passing the qualification examination for rural assistant GP

GP = general practitioner.

With the government’s great efforts, the trained and qualified GPs in China have increased from 109 794 in 2012 to 308 740 in 2018. Considering the essential goal of the establishment of general practice system and the regional disparities in development and health services, the rational and equitable allocation of GPs between different regions would be the key to better health service system reform. However, there are few studies focusing on the distribution of China’s GPs. This study compared and discussed the number of GPs and accessed the GPs’ distribution in China since the general practice system established, which was divided into three regions by their economic level and geographic position, to show the current equality in GPs in different regions of China and provide the references for optimization of PHC workforce system in China.

## Methods

### Setting

China is the largest developing country with a per capita gross national income (GNI) of disposable income of 10 937.98 USD (according to the exchange rate on October 1, 2021, accessed in https://treasury.un.org/) in 2019, and the population in China is about 1.4 billion in total. Over the past decades, China has experienced rapid demographic and epidemiological transitions with the economic boom (Zhou *et al.*, 2019). The data of the seventh national population census show that as of November 1, 2020, the number of people aged 60 and over in China had reached 264 million. There is a progressive shift in the burden of disease to chronic Non-Communicable Diseases (NCDs), such as Cardiovascular Diseases, Lung Cancer, Chronic Obstructive Pulmonary Disease (COPD), Diabetes, and Obesity (Yang *et al.*, 2013; Thomas *et al.*, 2020). The rapid rise of NCDs driven by urbanization, rising incomes, lifestyle changing, and ageing poses major challenges to China’s health system, which may be mitigated with a stronger PHC support system (Comino *et al.*, 2012; Badora-Musiał *et al.*, 2021). China has 34 provinces, autonomous regions, and municipalities, 31 of which are in the mainland. The measurement of inequality of GPs distribution mainly focuses on the mainland of China. Given the economic level and geographic position, the 31 provinces, autonomous regions, and municipalities were statistically divided into three divisions, the Eastern division (Beijing, Tianjin, Hebei, Liaoning, Shanghai, Jiangsu, Zhejiang, Shandong, Fujian, Guangdong, and Hainan), the Central division (Shanxi, Jilin, Heilongjiang, Anhui, Jiangxi, Henan, Hubei, and Hunan), and the Western division (Inner Mongolia, Guangxi, Chongqing, Sichuan, Guizhou, Yunnan, Tibet, Shaanxi, Gansu, Qinghai, Ningxia, and Xinjiang). According to the per capita disposable income of the provinces in different subregions in 2018 (Table 2), it is obvious that the per capita disposable income of residents in the provinces and municipalities in the Eastern division is higher, followed by the Central division, and the lowest in the Western division.

**Table 2.** Numbers and densities of general practitioners from 2012 to 2018

Region	**2018 disposable income (USD)** ^a^	Numbers							**Density (per 10** **000 population)**						
		2012	2013	2014	2015	2016	2017	2018	2012	2013	2014	2015	2016	2017	2018
**Eastern Division**		**66 401**	**84 464**	**96 979**	**104 015**	**116 537**	**139 473**	**170 362**	**1.19**	**1.5**	**1.71**	**1.83**	**2.03**	**2.42**	**2.93**
Beijing	9644.48	8137	8458	8221	8269	8402	8591	8861	3.93	4.00	3.82	3.81	3.87	3.96	4.11
Tianjin	6109.82	1095	1427	1622	2144	2403	3749	4138	0.77	0.97	1.07	1.39	1.54	2.41	2.65
Hebei	3626.00	3493	6730	8637	9286	9355	10 017	11 292	0.48	0.92	1.17	1.25	1.25	1.33	1.49
Liaoning	4593.47	3304	3513	3777	3624	4195	6273	9002	0.75	0.80	0.86	0.83	0.96	1.44	2.07
Shanghai	9926.17	5323	5957	6925	7352	7967	8491	8629	2.24	2.47	2.85	3.04	3.29	3.51	3.56
Jiangsu	5891.71	15 068	17 650	19 748	20 841	25 162	27 578	47 794	1.90	2.22	2.48	2.61	3.15	3.43	5.94
Zhejiang	7089.36	12 251	17 041	19 640	21 627	22 571	30 467	26 047	2.24	3.10	3.57	3.90	4.04	5.39	4.54
Shandong	4516.64	6775	7709	8967	9920	11 372	13 565	17 426	0.70	0.79	0.92	1.01	1.14	1.36	1.73
Fujian	5048.55	2594	3634	4310	5122	5786	6897	8182	0.69	0.96	1.13	1.33	1.49	1.76	2.08
Guangdong	5538.18	7940	11 765	14 404	14 955	18 338	22 712	27 638	0.75	1.11	1.34	1.38	1.67	2.03	2.44
Hainan	3801.27	421	580	728	875	986	1133	1353	0.47	0.65	0.81	0.96	1.08	1.22	1.45
**Central Division**		**22 192**	**29 674**	**39 020**	**45 344**	**49 944**	**63 269**	**75 302**	**0.52**	**0.70**	**0.91**	**1.05**	**1.16**	**1.46**	**1.73**
Shanxi	3400.88	2552	2958	3618	4014	4175	6372	5962	0.71	0.81	0.99	1.10	1.13	1.72	1.60
Jilin	3525.89	1231	1680	2299	2891	3384	5130	4965	0.45	0.61	0.84	1.05	1.24	1.89	1.84
Heilongjiang	3514.66	2081	2889	3730	4320	4454	4493	5637	0.54	0.75	0.97	1.13	1.17	1.19	1.49
Anhui	3709.19	3191	4319	6814	7360	8625	10 430	12 917	0.53	0.72	1.12	1.20	1.39	1.67	2.04
Jiangxi	3724.05	2081	2429	3020	3319	3641	5268	5620	0.46	0.54	0.66	0.73	0.79	1.14	1.21
Henan	3396.77	4722	6427	8394	10 349	12 129	15 567	20 497	0.50	0.68	0.89	1.09	1.27	1.63	2.13
Hubei	3992.34	3752	5044	6090	6970	7020	8969	10 863	0.65	0.87	1.05	1.19	1.19	1.52	1.84
Hunan	3903.60	2582	3928	5055	6121	6516	7040	8841	0.39	0.59	0.75	0.90	0.96	1.03	1.28
**Western Division**		**21 201**	**31 373**	**36 598**	**39 290**	**42 602**	**49 975**	**63 076**	**0.58**	**0.86**	**0.99**	**1.06**	**1.14**	**1.33**	**1.66**
Guangxi	3322.77	3087	4039	4527	4671	5104	6275	7958	0.66	0.86	0.95	0.97	1.05	1.28	1.62
Inner Mongolia	4388.45	1679	2374	2937	3085	3178	3986	4894	0.67	0.95	1.17	1.23	1.26	1.58	1.93
Chongqing	4080.70	1632	2187	2527	2872	3127	3866	6348	0.55	0.74	0.84	0.95	1.03	1.26	2.05
Sichuan	3473.65	4665	8983	9819	10 394	10 360	11 343	13 404	0.58	1.11	1.21	1.27	1.25	1.37	1.61
Guizhou	2850.32	1032	1511	2416	3147	3714	5014	6238	0.30	0.43	0.69	0.89	1.04	1.40	1.73
Yunnan	3106.12	3212	4261	4106	4289	4737	5253	6381	0.69	0.91	0.87	0.90	0.99	1.09	1.32
Tibet	2673.38	34	67	109	161	202	247	352	0.11	0.21	0.34	0.50	0.61	0.73	1.02
Shaanxi	3484.12	1824	1978	2770	2126	2738	3578	4979	0.49	0.53	0.73	0.56	0.72	0.93	1.29
Gansu	2704.67	1389	2106	2710	3312	3773	3824	4835	0.54	0.82	1.05	1.27	1.45	1.46	1.83
Qinghai	3210.22	462	758	881	961	993	1230	1315	0.81	1.31	1.51	1.63	1.67	2.06	2.18
Ningxia	3464.34	260	392	471	565	654	926	1279	0.40	0.60	0.71	0.85	0.97	1.36	1.86
Xinjiang	3325.12	1925	2717	3325	3707	4022	4433	5093	0.86	1.20	1.45	1.57	1.68	1.81	2.05

a Calculated according to the exchange rate on October 1, 2021 (https://treasury.un.org/).

### Data

For the GPs system was established in 2011 to improve the health service capability at the grass-roots level, the study chose the data of Chinese GPs from 2012 to 2018 to measure the inequality of their demographical distribution. The research extracted the national data of GPs and analyzed the inequality of their distribution in China. The main data sources for this analysis were collected from *China Health Statistical Yearbook 2013–2019* (National Health Commission of the People’s Republic of China, 2014, 2015, 2016, 2017, 2018, 2019b, 2020). National Health Commission recorded all the basic health-related data in China, such as health institutions, health human resources, health equipment, health service, health expenditure, public health, health security, etc. The data of GPs were mainly contained in the health human resources.

### Measures of inequality

The descriptive analysis was conducted to show the overall changes in the number of GPs in China and changes in the three divisions and lower-level regions and the densities per 10 000 persons across the divisions. Then, the Gini coefficient and Theil L index were chosen to investigate the inequality trends in the densities of GPs. The Gini coefficient was calculated mathematically based on the Lorenz curve (Yitzhaki, 1979). In this study, the Gini coefficient was calculated according to the demographical distribution of GPs. The Gini coefficient has four levels for its value: the Gini coefficient < 0.2 indicates absolute equality, 0.2–0.3 relative equality, 0.3–0.4 proper inequality, and above 0.4 represents severe inequality (Zhou and Qin, 2012). Unlike the Gini coefficient, the Theil index could explain whether inequality mainly comes from between or within the divisions. The Gini coefficient and Theil L index both took the values between 0 and 1, with higher values indicating higher levels of inequality (Litchfield, 1999). The Gini coefficient and Theil L index are widely adopted as indicators to investigate the inequality, but the Gini coefficient cannot be decomposed to explain the sources of the inequalities. The Theil L index has the advantage of decomposition, which means decomposing the total national inequality to inner-regional difference and inter-regional difference (Anand, 2010; Zhou *et al.*, 2015; Ren *et al.*, 2018). More details about the methods could be seen in the authors’ another research (Yang, Wang, and Xue, 2019).

The Lorenz curve and Gini coefficient were performed in Microsoft Excel 2019 (Microsoft Corporation, Redmond, WA, USA). The Theil L index was calculated in MATLAB R2016a (The MathWorks Inc., Natick, MA, USA).

## Results

The descriptive statistics of GPs in China from 2012 to 2018 with the total number and densities per 10 000 population at the divisional level are shown in Table 2. The number of GPs showed an increasing trend. In the Eastern division the GPs increased from 66 401 in 2012 to 170 362 in 2018, in the Central division it grew from 22 192 in 2012 to 75 302 in 2018, and in the Western division it rose from 21 201 in 2012 to 63 076 in 2018.

Generally, the GPs density in China showed a fast growth from 2012 to 2018. However, there was a gap in the GPs densities between different divisions in China. In 2018, there were 2.93 GPs per 10 000 population in the Eastern division, but just 1.73 GPs per 10 000 population and 1.66 GPs per 10 000 population in the Central division and the Western division, respectively. The GPs density in the Central division in 2018 was 3.33 times more than that in 2012, showing a faster growth rate than the other two divisions. In the Eastern division, there were gaps between different subregions, such as the Beijing-Tianjin-Hebei region, also known as the Jing-Jin-Ji (JJJ) metropolitan region. The GPs per 10 000 population was 1.49 in Hebei province in 2018, which was much lower than that in nearby Beijing (4.11 GPs per 10 000 population) and Tianjin (2.65 GPs per 10 000 population).

**Figure 1.**
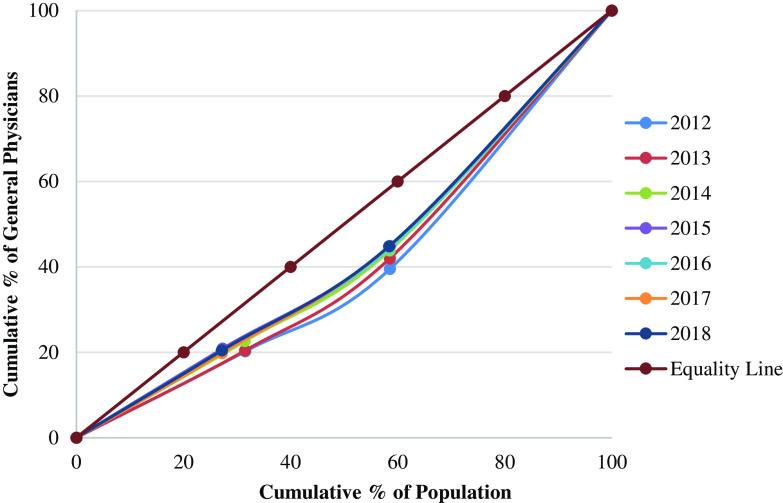
Lorenz curve showing the distribution of general practitioners according to population size at the divisional level

The Lorenz curve in Figure 1 shows the cumulative share of GPs against the cumulative share of population from 2012 to 2018. Figure 1 shows that at the divisional level the GPs remained not that flat but close to the equality line, which indicated the rather good equality in the GPs distribution in China. The Gini coefficient (Table 3) of the GPs declined from 0.234 in 2012 to 0.167 in 2018, which means the equality in GPs turned from rather fair into perfectly fair.

**Table 3.** Inequality in general practitioners across divisions and districts

Year	Gini coefficient	Theil L index for each division			Within divisions (%)	Across divisions (%)	Theil L index
		Eastern division	Central division	Western division			
2012	0.234	0.165	0.014	0.055	0.233 (63%)	0.139 (37%)	0.372
2013	0.210	0.154	0.015	0.073	0.242 (68%)	0.112 (32%)	0.354
2014	0.181	0.148	0.018	0.062	0.228 (69%)	0.101 (31%)	0.329
2015	0.167	0.141	0.020	0.059	0.220 (69%)	0.097 (31%)	0.317
2016	0.173	0.148	0.023	0.052	0.223 (68%)	0.102 (32%)	0.325
2017	0.170	0.148	0.022	0.045	0.214 (67%)	0.104 (33%)	0.318
2018	0.167	0.172	0.030	0.044	0.246 (71%)	0.099 (29%)	0.345

Table 3 shows the decomposition of inequality in GPs according to the results of Thiel L index. The Thiel L index, fluctuating from 0.372 in 2012 to 0.345 in 2018, reflected rather inequality concerning the differences between divisions and within divisions. The inequality in the GPs distribution within divisions contributes more to the overall inequality than the inequality between divisions. For the inequality within divisions, the Eastern division’s Theil L index was higher than that of other divisions, indicating that even though the general number and the density of GPs in the Eastern division was at the forefront, the inequality in GPs distribution between the provinces and municipalities in the Eastern division was more serious.

## Discussion

### Achievements and shortage of GPs in China

As part of China’s healthcare reform effort to provide people with equitable, accessible, affordable, and effective health care, PHC has received considerable attention. Strengthening PHC is the focus of the healthcare reform and the basic way to improve the fairness, accessibility, and affordability of basic medical and health services. However, evidence suggests that the quality of PHC in China is still suboptimal (Li *et al.*, 2017). The lack of PHC doctors and the low education and qualification among PHC professionals block the improvement of quality and ability of PHC (Li *et al.*, 2020). This situation may lead to two phenomena which were common in China, for people who were rich and accessible to large hospitals, they would prefer to go to large hospitals directly, even if they only suffer from some ailments (Mathers and Huang, 2014; World Bank Group, 2016; Jiang *et al.*, 2020), which caused the congestion of patients in large hospitals, reduced the efficiency of medical resource utilization, increased the pressure on health workers of big hospitals, and even worsened the conflict between doctors and patients. For people who were poor and less educated, they might not go to the doctor, due to insufficient attention to the disease and lack of qualified basic medical resources, eventually leading to the deterioration of their health (Huang *et al.*, 2010; Guo *et al.*, 2020). More equitable health resources and access can help people get rid of poor health and poverty caused by disease. GPs play an important role in protecting people’s health as the gatekeeper, especially for the poor people living in impoverished areas lacking medical resources. Since the GPs system established, the number of GPs in China had been almost tripled (from 109 794 in 2012 to 308 740 in 2018), and the GP system improved the efficiency and quality of PHC (Li *et al.*, 2018; Liang *et al.*, 2019). However, although the number of GPs in China had been increasing rapidly since the GP system built, the qualified GPs still constituted only a small proportion (from 4% in 2011 to 13% in 2018) of all doctors practicing in China’s PHC settings (Li *et al.*, 2020). According to *China Health Statistics Yearbook*, the number of health care institutions at the grass-roots level had increased from 328 824 in 2012 to 353 895 in 2018 in the Eastern division, but only slightly increased from 288 406 to 295 189 in the Western division. Moreover, considering the threshold of 2–3 GPs per 10 000 population in 2020 issued by the State Council of China, most regions of the Central and Western divisions had not met it, far below the health needs of people there, and the whole nation still faced a severe shortage of GPs (Wu, 2018). Without enough GPs, people living in the Central and Western division may have worse health condition due to the lower PHC accessibility, the relatively backward social and economic development, and the rather lower health literacy.

### Inequality in GPs’ distribution

The Gini coefficient showed the high equality in the GPs distribution in China. However, the shortcomings of GPs’ distribution were also revealed in this study, such as the rather low number of GPs in some provinces, autonomous regions, and municipalities, and most of them were less developed and belonged to the Central and Western divisions. The GP-to-population ratio of the Central division was the lowest in 2012, and in 2018 that of the Western division became the lowest. Given that there were fewer population in the Western division, the relatively small number of GPs seemed to be able to meet the health service needs of the local people. However, that’s not the case. Several studies found the geographic distribution of health care resources in China exhibits a high level of inequality (Jin *et al.*, 2015; Wu and Yang, 2019). The geographical area of the Western China was larger, since the service coverage of GPs was limited, and the small number of GPs could lead to the low accessibility of health services, especially for those scattered people living in remote areas. Due to the poor economic development, allowance policy, and living and education conditions, for example, Fujian Province of the Eastern division provided the allowance of about 4639.65 USD/year for each trainee of assistant GP training programme, but Sichuan province of the Western division provided the allowance of about 2474.48 USD/year for each trainee of assistant GP training programme, the Western areas of China was relatively disadvantaged in terms of resource inflow, talent introduction and talent training, and the needs to strengthen the PHC system and people’s health there was more urgent. Hence, the increase of the number of GPs and the improvement of GPs’ health service capacity in the Central and Western areas remain the key points of Chinese health system. Considering the level of socio-economic development will affect the flow and allocation of health resources, the Central and Western regions not only need to make more efforts to optimize the training and financial investment of GPs system, but also need to think about how to promote local socio-economic development, so as to support the implementation of the GPs system.

In addition to the GPs densities in different divisions, the Theil L index results indicated the inequality in GPs distribution in China mainly came from the differences within divisions. The inequality in GPs within division (i.e., across provinces of each different division) contributed about 63% in 2012 to 71% in 2018 to the total Theil L index, which showed a rising trend and meant the gap was expanded. The inequality in GPs of the Eastern division was most outstanding, and then the Western division. When it comes to the health inequality, the inequality in those poor and remote areas always attracts more attention. However, in this study, the results showed that the inequality between and within less developed and developed areas should both be paid more attention. The inequality in health resources in the Eastern division has been found in several studies. For the JJJ metropolitan regional development imbalance, the limited medical education resources and the large gap in socio-economic development, such as per capita disposable income of residents between Hebei (3626.00 USD) and Beijing (9644.48 USD) and Tianjin (6109.82 USD), and more medical schools included in the 211 and 985 projects in Beijing and Tianjin, weaken its advantages in recruitment, training capacity, and the number of general practice students and graduates. At the same time, Hebei is also at a disadvantage in the competition with Beijing and Tianjin for medical talent recruitment. In China, it takes the medical students about eight years to be a licensed GPs under the “5 + 3 GPs training program.” After a long time of learning and training, the GPs would prefer the big cities, like Beijing and Tianjin, to Hebei under the context that Beijing and Tianjin have better economic, educational, and occupational conditions. This may further aggravate this inequality situation (Zhou *et al.*, 2015). Under such circumstances, people who live in less-developed areas still have poor accessibility to health services without effective health policies and interventions to change the flow of health workforce.

### Health labor market lens and policy implications

As a developing county, general practice services in China are still in an early stage of development (Zhang *et al.*, 2019). In China, the GPs system is established for narrowing that gap of health service accessibility between urban and rural areas and the rich and the poor, improving the health service quality and capacity at the grass-root level, and protecting the people’s health universally. The GPs system is important to improve the public health service system, strengthen the construction of grassroots prevention, and control capabilities in rural areas and communities. Given the imbalanced medical resources distribution and socio-economic development between the Eastern division and the Central and Western division and between the urban areas and the rural and remote areas, a more adequate GPs supply and balanced GPs distribution are important to improve Chinese health system and people’s health, especially for the Central and Western divisions and the rural and remote areas, where the health resources and people’s health status are relatively poor. A more equitable GPs distribution is definitely good for disease reduction and health equity, including universal health coverage (UHC). However, the GPs are relatively lacking and currently structurally imbalanced, for example, the Central and Western regions have a small number of GPs, and most of the existing GPs are concentrated in communities of cities and towns and few of them would like to work in the villages. There are many factors causing the shortage of GPs and the inequality in their distribution, such as salary, career prospects and promotion, living condition, and job satisfaction (Wang *et al.*, 2013; Mathers and Huang, 2014; Lian *et al.*, 2019; Zhu and Ariana, 2020). From the perspective of health labor market, the health labor market would be “clear” when the supply of labor matches the demand for it, but labor markets do not always “clear” in this way, and there are often situations where graduates cannot find a satisfactory job or labor shortage (McPake *et al.*, 2013). A health labor market is a dynamic system that needs to be analyzed from both the demand and the supply of health workers. The demand for health workers is influenced by people’s need for health care and the government or the medical institutions’ willingness to hire them, and the supply of qualified heal workers is determined by many factors, such as the number of graduates, training system, financial and non-financial incentives, and working environment (Chen *et al.*, 2004; McPake *et al.*, 2013; Liu *et al.*, 2017). There are a number of reasons for an imbalance between the demand and supply for health workers, for example, price (i.e., wages or “compensation”) may not be easily adjusted due to the regulations established by legislative or bureaucratic process. Thus, a better health labor market needs the government or institution to take more effective interventions to achieve better health performance. In China, the government has issued many interventions, such as licensing assistant GP and GP, encouraging qualified universities to establish general medicine teaching and research sections, general medicine departments or general medicine schools, subsidizing GP education, and GPs working in rural and remote areas, cultivating rural-origin tuition-waived medical students to direct more health workers to rural and remote areas. With these efforts, the number of GPs in China has increased significantly, increasing by 2.81 times from 2012 to 2018. However, the imbalanced distribution is still a problem we need to pay attention to, and we should take both supply and demand constraints into consideration simultaneously. It is important to improve the ability to train GPs in the Central and Western divisions, strengthen the GP professional training capabilities of medical schools and disciplines there, and build a more complete GPs training system. Meanwhile, more incentive policies are needed to be made to recruit and retain GPs in the Central and Western divisions, including remuneration, professional promotion, living condition for GPs and their family members, and other compensations. More incentive policies should be made to attract GPs to work in the Western China and the rural and remote areas in China. Increasing the rural-origin tuition-waived medical students would be a good way (Matsumoto, Inoue, and Kajii, 2008), and a general practice system offering more guaranteed income and promising career promotion and professional development is also necessary (Mathers and Huang, 2014).

The Chinese government needs to develop more effective policies for the increase in the total number of GPs and the equitable and rational allocation of GPs. Considering the current situation of China, it is believed that the imbalanced development of socio-economic levels in different regions, long period of upfront time and effort investment, less incentive policies (low salary, heavy workloads, and low job satisfaction) and supportive policies (living condition, education condition for themselves and their children) are the possible causes for the GPs inequality distribution in China. With the changes in disease spectrum and population structure, the increasing demand on high-quality and accessible health service, the GP-based PHC needs to be further improved in quantity, quality, and structure in the future. The Chinese government should not only narrow the gap between the urban and rural areas and the Eastern and the Western areas, but also balance the GPs distribution between divisions and within divisions according to the population, health needs, and geographical area (Matsumoto *et al.*, 2010). For those areas with poor educational condition and other conditions conducive to GPs training and retention, the central government and local government could make more comprehensively supportive policies, such as the e-learning and training program, the economic development and GPs collaboration plan between neighboring provinces and cities.

### Limitations of this study

There are some limitations in this study. Given China’s present situation, the GP-based PHC implemented in China is the main measure to improve the accessibility and quality of basic medical and health care services, therefore, the study focused on the change and equality of GPs in China and did not analyze other PHC doctors. The GPs density and population data were directly used from the health statistics yearbook, considering the survey design, and the inequality was analyzed at the national and regional level, and deeper discussion about the source of provincial inequality and other potential influencing factors of inequality will be furthered. In this study, the Gini coefficient was used to analyze the inequality of demographical distribution of GPs, and the migrant population was not included in the analysis due to lack of data, hence the accuracy of our analysis on the inequality of GPs’ distribution would be compromised. Further studies about health labor (GPs) market analysis considering the market structure are needed to reveal the detailed sources of inequality and to provide evidence for national and local policymaking.

## Conclusions

This study assessed the inequality in GPs distribution in China from 2012 to 2018 by Lorenz curve, Gini coefficient, and Theil L index, given the critical role of GPs played in PHC service system. According to the results, the number of GPs showed a fast growth in recent years, but the shortage and maldistribution of GPs should not be neglected. The inequalities across provinces, autonomous regions, and municipalities were the major source of inequality in GPs distribution. Findings from this study indicated the shortage and maldistribution of GPs in China still needs more efforts to alleviate, especially pay more attention to those remote and less developed areas. The Chinese government should take the findings of this study into consideration in the future GPs-related policy-making and further improve the equitable allocation of health resources to supply the more affordable, accessible, and appropriate PHC. For rural and remote areas in China, the health administration department at the grass-roots level should strengthen the community’s basic and bridging role in GP training and establish more supporting policies for community teaching bases. Meanwhile, the Chinese government should gradually reform and implement an effective and reasonable price system (wage and “compensation”) to provide guarantee for the development of the GP system, especially in the Central and Western divisions.

Further studies are needed to explore the health service utilization of GPs, reveal the deep reasons for the inequality, and assess the role of general practice system in improving health inequality caused by inequality in socio-economic development to provide evidence for national and local policy-making.
